# Inhaled Long-Acting β2-Agonists Do Not Increase Fatal Cardiovascular Adverse Events in COPD: A Meta-Analysis

**DOI:** 10.1371/journal.pone.0137904

**Published:** 2015-09-17

**Authors:** Ning Xia, Hao Wang, Xiuhong Nie

**Affiliations:** 1 Department of Respiratory Diseases, Xuanwu Hospital, Capital Medical University, Beijing, China; 2 Department of Cardiology, Fuwai Hospital, National Center for Cardiovascular Diseases, Chinese Academy of Medical Sciences and Peking Union Medical College, Beijing, China; Bascom Palmer Eye Institute, University of Miami School of Medicine;, UNITED STATES

## Abstract

**Background:**

The cardiovascular safety of inhaled long-acting β_2_-agonists (LABAs) in patients with chronic obstructive pulmonary disease (COPD) is a controversial problem. Certain studies have suggested that inhaled LABAs lead to an increased risk of cardiovascular events in patients with COPD. This meta-analysis aimed to assess the cardiovascular safety of inhaled LABAs in COPD.

**Methods:**

A meta-analysis of randomized, double-blind, parallel-group, placebo-controlled trials for LABA treatment of COPD with at least 3 months of follow-up was performed. The fixed-effects model was used to evaluate the effects of LABAs on fatal cardiovascular adverse events. Adverse events were collected for each trial, and the relative risk (RR) and 95% confidence intervals (CI) for LABA/placebo were estimated.

**Results:**

There were 24 trials included in this meta-analysis. Compared with placebo, inhaled LABAs significantly decreased fatal cardiovascular adverse events in COPD patients (RR 0.65, 95% CI 0.50 to 0.86, P = 0.002). In sensitivity analysis, there was still no increased risk of fatal cardiovascular events (RR 0.68, 95%CI 0.46 to 1.01, P = 0.06) after excluding the trial with the largest weight. Among the different types of LABAs, only salmeterol had a significant effect (RR 0.64, 95% CI 0.46 to 0.90). In subgroup analyses, inhaled LABAs were able to significantly decrease fatal cardiovascular events in long-term trials (RR 0.64, 95% CI 0.47 to 0.87) and in trials with severe COPD patients (RR 0.69, 95% CI 0.50 to 0.96).

**Conclusion:**

Inhaled LABAs do not increase the risk of fatal cardiovascular events in COPD patients.

## Introduction

Chronic obstructive pulmonary disease (COPD) is a common respiratory disorder that leads to high morbidity and mortality. Worldwide, COPD affects 329 million people or nearly 5% of the population [1]. Certain common symptoms of COPD, such as dyspnea, chronic cough, and shortness of breath, reduce the patients’ quality of life and cause a serious economic burden on healthcare resources. Inhaled bronchodilators, including β_2_-adrenoceptor agonists and anticholinergics, play a significant role in treating COPD. Inhaled β_2_-adrenoceptor agonists relieve the symptoms of COPD by relaxing the bronchial smooth muscles. Short-acting β_2_-adrenoceptor agonists (SABAs) are recommended on an as needed basis, whereas long-acting β_2_-adrenoceptor agonists (LABAs) are recommended as a first-line therapy for controlling the symptoms of COPD. Several meta-analyses have provided evidence that LABAs significantly reduce COPD exacerbations, improve pulmonary function and quality of life and are well tolerated [2–4].

However, COPD is often accompanied by cardiac disorders, and a proportion of the deaths of patients with COPD are the result of cardiovascular complications [5]. It has been reported that reduced FEV1 among COPD patients nearly doubles the risk of cardiovascular mortality, independent of age, sex and cigarette smoking [6].Thus, it is important to evaluate the cardiovascular safety of medications for treating COPD. Given that β_2_-receptors are also present in the heart, which can cause cardiac toxicity [7], the cardiovascular safety of using inhaled LABAs in COPD patients remains a controversial issue. Certain evidence has showed that inhaled LABAs increase the risk of adverse cardiovascular events [8, 9]. Salpeter et al performed a meta-analysis and found that compared with placebo, inhaled LABAs significantly increased sinus tachycardia, with a non-significant trend toward an increase in major cardiovascular events, including atrial fibrillation, congestive heart failure and myocardial infarction [8]. In contrast, Ferguson et al [10] analyzed seven placebo-controlled trials and provided evidence that there was no significant difference between salmeterol (a type of LABA) and placebo in terms of cardiovascular events and deaths. Moreover, a large double-blind, parallel-group, placebo-controlled trial with a 3-year follow-up, known as the TORCH study, found no significant difference between salmeterol and placebo with respect to cardiovascular complications [11]. Considering the evidence mentioned above, it is still uncertain whether inhaled LABAs can increase cardiovascular events and death in COPD patients. Moreover, certain new once-daily LABAs, such as indacaterol, olodaterol and vilanterol, have recently shown efficacy in improving COPD symptoms and reducing exacerbations [12–18], but the influence of these new agents on cardiovascular events is unknown. Therefore, we conducted this meta-analysis to determine the effect of inhaled LABAs compared with placebo on fatal cardiovascular events in COPD patients.

## Methods

### Eligibility criteria

The inclusion criteria for trials were as follows: (1) the study was a randomized controlled trial(RCT) with a double-blind, parallel-group, placebo-controlled design and more than 3 months of follow-up including any inhaled LABA; (2) the study patients had COPD of any severity; (3) the trial provided the details of fatal cardiovascular events and reported at least one such event; (4) trials were excluded if they enrolled patients with asthma, did not compare a LABA with placebo, were only protocols or abstracts, or were written in non-English languages.

### Search strategy

We searched MEDLINE, EMBASE, the Cochrane Library and Clinicaltrials.gov for trials. We also examined the bibliographies of related systematic review articles for eligible trials, and two investigators independently reviewed all trials to determine if each study was an RCT and met the inclusion criteria mentioned above. The search strategy was “long acting beta 2 agonist” OR “long acting beta agonist” OR “LABA” OR “adrenergic beta 2 receptor agonists” OR “adrenergic beta agonists” OR “bronchodilator agents” OR “salmeterol” OR “formoterol” OR “olodaterol” OR “vilanterol” OR “indacaterol”, and “chronic obstructive pulmonary disease” OR “chronic obstructive lung disease” OR “chronic obstructive airway disease” OR “pulmonary disease, chronic obstructive” OR “COPD”, based on a previously published meta-analysis [19].

### Outcome measures and data extraction

The primary outcomes were fatal cardiovascular adverse events. All events were coded using the Medical Dictionary for Regulatory Activities (MedDRA) [20]. We defined fatal cardiovascular events to include fatal events reported in the trials and three other terms (not coded as cardiovascular events by the MedDRA), which were sudden death, sudden cardiac death and cardiac death, as described in a previous meta-analysis [21]. Two reviewers independently and separately extracted the outcome data, and a third reviewer provided additional insight in the event of a discrepancy. If the primary outcomes were not available in the original articles, we searched for details using Clinicaltrials.gov, the US FDA website, and the manufacturers’ clinical trials registry website. All trials were evaluated using the Jadad scoring system, and a score >2 was required for a trial to be kept in the analysis [22].

### Statistical analysis

Review Manager (RevMan) version 5.0 was used to calculate the relative risk (RR) and 95% confidence intervals (CIs) for the primary outcomes. A two-sided α value of 0.05 was defined as statistically significant. Statistical heterogeneity was addressed using the Cochrane Q test, and the magnitude of heterogeneity was estimated by I^2^ statistic [23], where a value more than 50% indicates a substantial level of heterogeneity. We used the fixed-effects model to pool data if substantial statistical heterogeneity was not present. Visual inspection of funnel plots was used to assess the presence of publication bias.

Subgroup analyses were carried out with the data segregated by the different types of inhaled LABAs, the study duration (≤6 months vs. >6 months), and the severity of COPD (FEV1>50% vs. FEV1≤50%). Sensitivity analysis was performed to evaluate the influence of individual studies on the summary effect by looking at the influence of each study and then repeating the analysis after excluding the study with the largest weight.

## Results

### Characteristics of the identified studies

**Fig 1:**
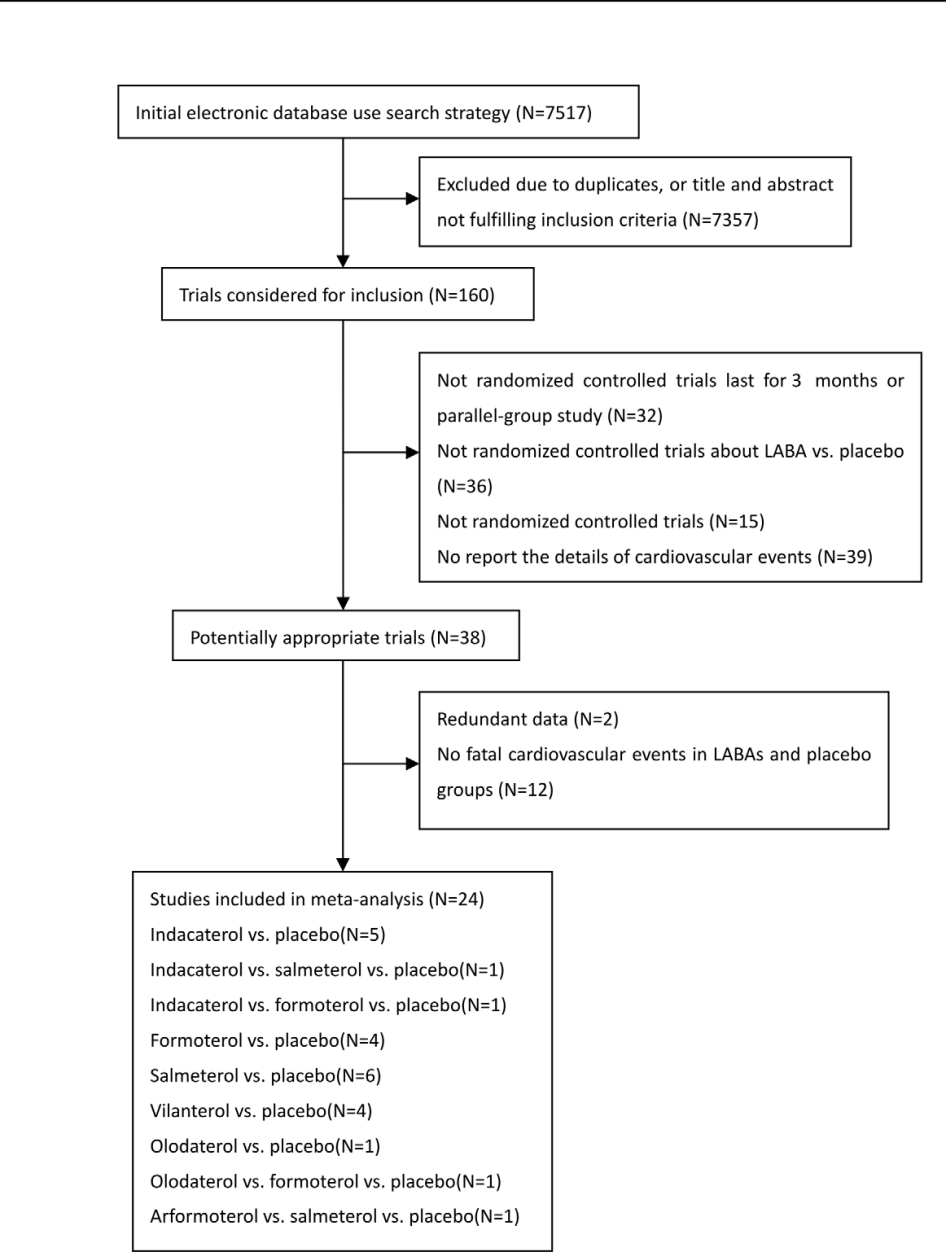
Flowchart of study identification

After a detailed review of 160 studies, a total of 24 trials that fulfilled the inclusion criteria were selected for the meta-analysis [11, 12, 14–18, 24–40]. The details of the trial selection strategy are shown in Fig 1, and the main characteristics of the included trials are shown in S1 Table. All trials were of good quality (Jadad score >2). In the 24 trials, a total of 12,291 patients received an inhaled LABA, and 7784 received placebo therapy. The mean age of the participants was more than 60 years in all of these trials. There were 8 long-term trials (longer than 6 months), and 16 short-term trials (12 to 26 weeks). The mean predicted FEV1 of the participants was more than 50% in 10 trials and less than 50% in 12 trials; 2 trials did not report the details of FEV1.

### Meta-analysis

**Fig 2:**
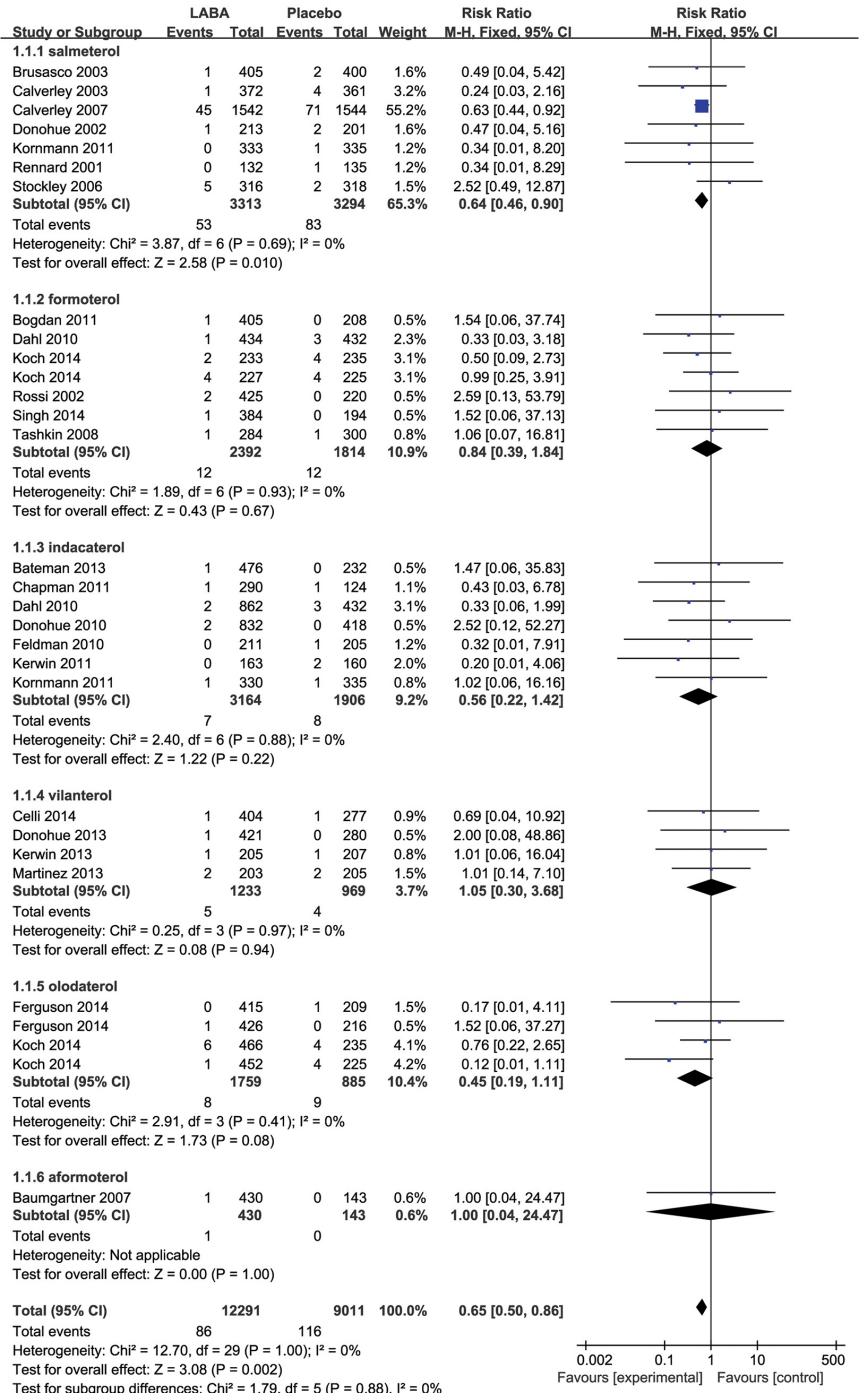
Meta-analysis of fatal cardiovascular events in RCTs of LABAs compare with placebo.

In total, 24 studies reported at least one fatal cardiovascular event. Compared with placebo, the inhaled LABAs significantly decreased the risk of fatal cardiovascular events in COPD (RR 0.65, 95% CI 0.50 to 0.86, P = 0.002, Fig 2) with no evidence of statistical heterogeneity (I^2^ = 0%, P = 1.00).

### Subgroup analyses

**Fig 3:**
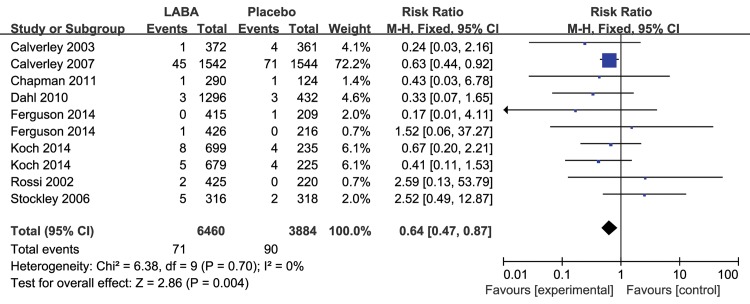
Meta-analysis of fatal cardiovascular events in long-term RCTs of LABAs compared with placebo.

**Fig 4:**
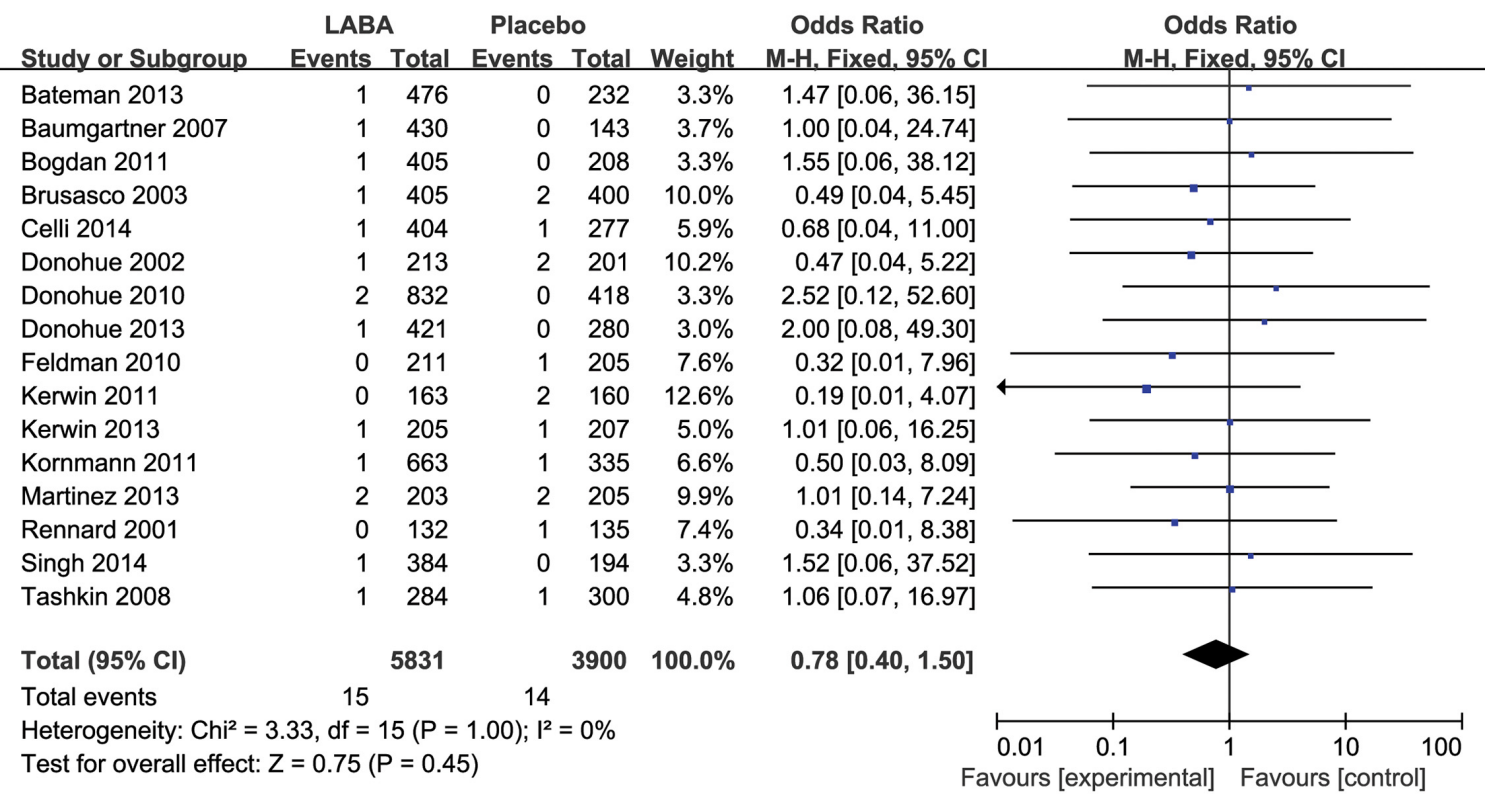
Meta-analysis of fatal cardiovascular events in short-term RCTs of LABAs compared with placebo.

**Fig 5:**
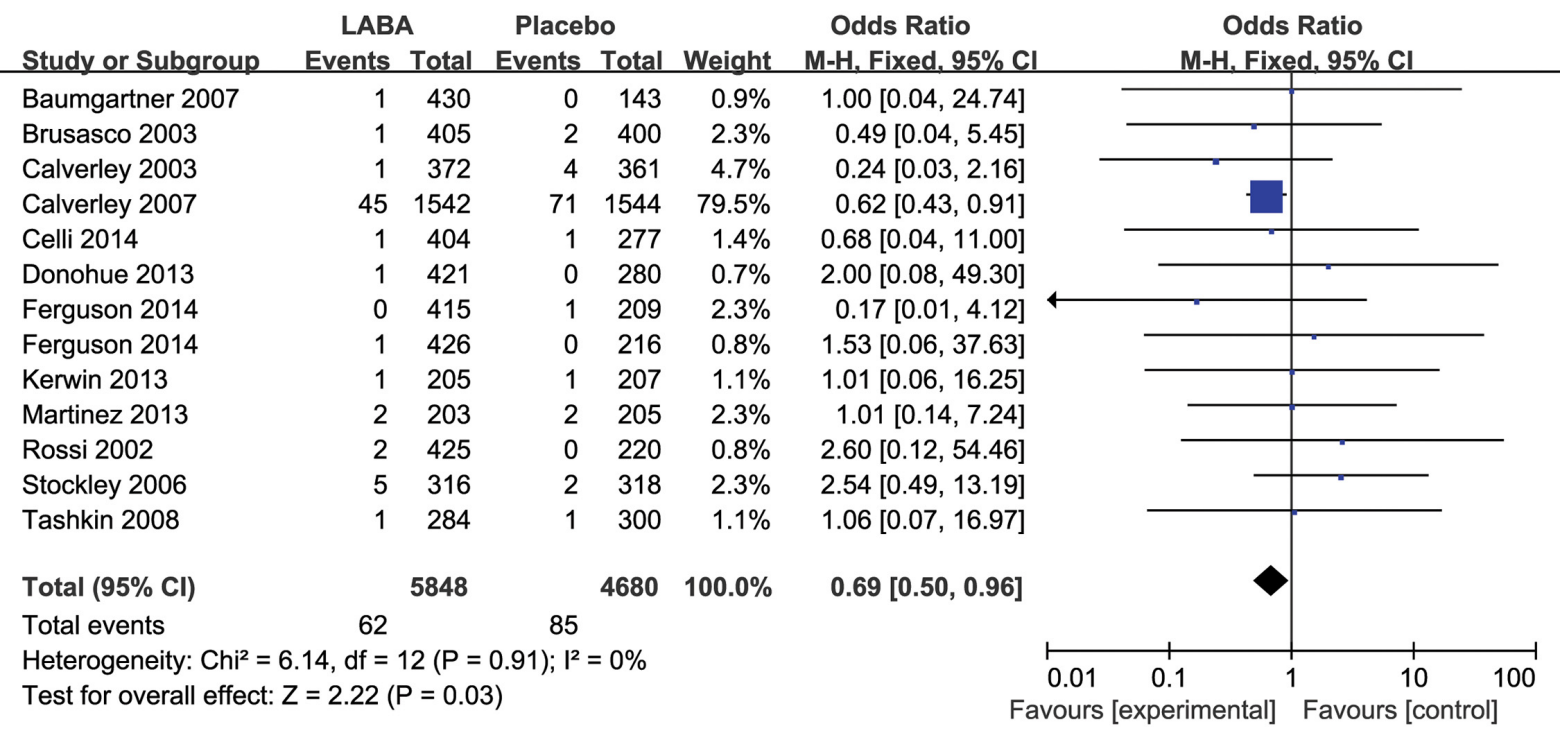
Meta-analysis of fatal cardiovascular events in RCTs (FEV1<50%) of LABAs compared with placebo.

**Fig 6:**
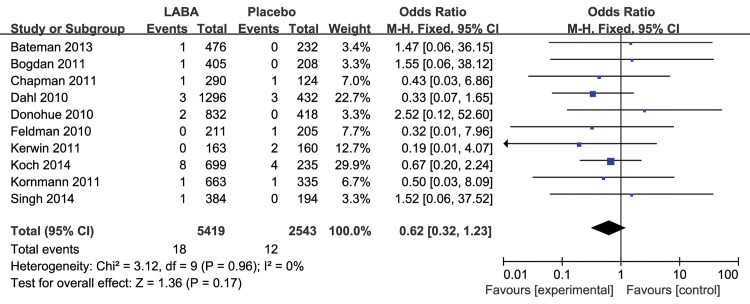
Meta-analysis of fatal cardiovascular events in RCTs (FEV1≥50%) of LABAs compared with placebo.

The results for the impact of different inhaled LABAs on fatal cardiovascular events are shown in Table 1. Moreover, there was a significant difference between the long-term and short-term trials. In the 8 long-term trials, LABAs significantly decreased the risk of fatal cardiovascular events compared with placebo (RR 0.64, 95% CI 0.47 to 0.87, P = 0.004, Fig 3), without obvious heterogeneity (I^2^ = 0%, P = 0.70). However, there was no significant difference between inhaled LABAs and placebo in terms of fatal cardiovascular events in the short-term trials (RR 0.78, 95% CI 0.40 to 1.50, P = 0.45, Fig 4). Furthermore, compared with placebo, LABAs significantly decreased the risk of fatal cardiovascular events in the trials in which the predicted FEV1 of the participants were less than 50% (RR 0.69, 95% CI 0.50 to 0.96, Fig 5). In contrast, there was no significant difference between inhaled LABAs and placebo in terms of fatal events in the trials in which the predicted FEV1 of participants were more than 50% (RR 0.62, 95% CI 0.32 to 1.23, Fig 6).

**Table 1:** Results of Meta-analysis of different inhaled LABAs on fatal cardiovascular events.

LABAs	No. of RCTs	No./Total No.		RR (95%CI)	P Value	I^2^,%^a^
		LABA	Controls			
Salmeterol	7	53/3313	83/3294	0.64(0.46,0.90)	0.01	0
Formoterol	7	12/2392	12/1814	0.84(0.39,1.84)	0.67	0
Indacaterol	7	7/3164	8/1906	0.56(0.22,1.42)	0.22	0
Vilanterol	4	5/1233	4/969	1.05(0.30,3.68)	0.94	0
Olodaterol	4	8/1759	9/885	0.45(0.19,1.11)	0.08	0
Aformoterol	1	1/430	0/143	1.00(0.04,24.47)	1.00	NA

Abbreviations: CI, confidence interval; RCT, randomized controlled trials; RR, relative risk

### Sensitivity analysis and publication bias

**Fig 7:**
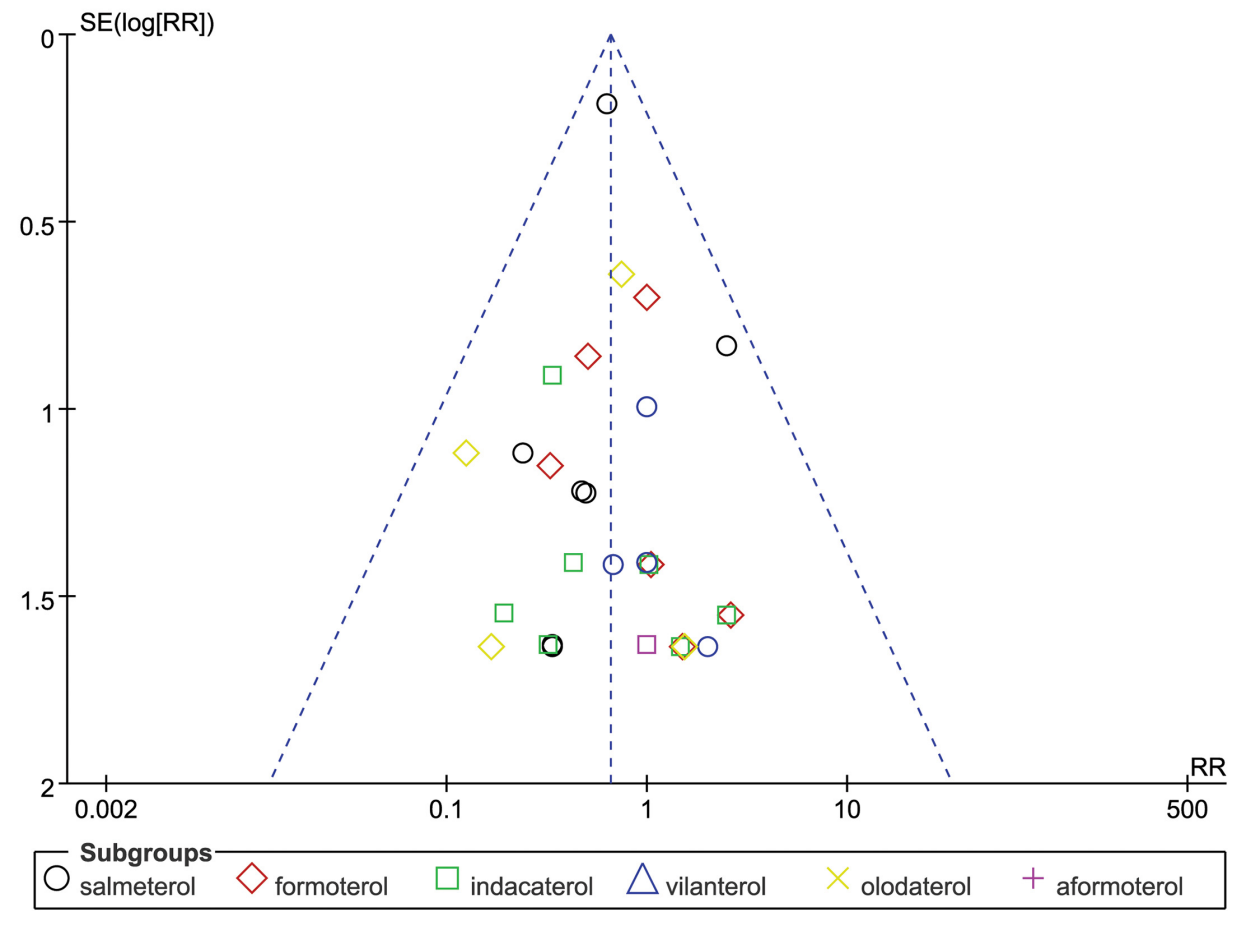
Funnel plot for studies reporting fatal cardiovascular events in COPD patients

We performed the leave-one-out sensitivity analysis by removing one study per time to check whether individual study influenced the results. After excluding the trial that contributed to more than 50% of the weight in the fixed-effects model (with the largest sample size and the longest duration of follow-up) [11], the analysis of the remaining 23 trials on the primary outcome of fatal cardiovascular events yielded effect sizes (RR 0.68, 95% CI 0.46 to 1.01, P = 0.06), which still did not increase the risk of fatal cardiovascular events. There was no evidence of statistical heterogeneity among the trials (I^2^ = 0%). Moreover, we used the funnel plot to assess the publication bias. The funnel plot was symmetrical, and no publication bias was found (Fig 7).

## Discussion

Based on the results of our meta-analysis of 24 clinical trials, inhaled LABAs significantly reduced the rate of fatal cardiovascular events in COPD patients compared with placebo (RR 0.65, 95% CI 0.50 to 0.86, P = 0.002). Moreover, the subgroup analyses showed that inhaled LABAs were able to significantly decrease fatal cardiovascular events in long-term trials and in studies with severe COPD patients (study duration >6 months and predicted FEV1 ≤50%).

There are certain potential mechanisms underlying the benefits of LABA treatment in terms of cardiovascular events. First, from a clinical perspective, it is known that COPD is an important cause of death and is closely related to cardiovascular morbidity and mortality [41]. There is certain evidence that recurrent COPD exacerbations, impairment of lung function, and respiratory failure are risk factors for fatal cardiovascular events, including malignant arrhythmia, ischemic heart disease, myocardial infarction and stroke [42–44]. In this context, it is important that the strategy for the treatment of COPD includes approaches to improve lung function and to reduce the frequency of exacerbations because these approaches can decrease cardiovascular morbidity and mortality. Numerous clinical trials have shown that inhaled LABAs significantly improve lung function and decrease the rate of COPD exacerbations compared with placebo, which might explain the observed outcome of favorable effects of LABAs. This finding also supported the results of the subgroup analyses. In long-term trials, the longer that inhaled LABAs were used, the more lung function improved. Therefore, the incidence of cardiovascular events decreased more significantly than in short-term trials. Moreover, as mentioned above, impairment of lung function was a risk factor for cardiovascular events in COPD patients. In trials with severe COPD patients (predicted FEV1≤50%), the rate of cardiovascular events was much higher than in trials with non-severe COPD patients (predicted FEV1>50%). Therefore, compared with placebo, the effect of inhaled LABAs on reducing cardiovascular events was more significant in trials with severe COPD patients. Second, with respect to biological mechanisms, it is known that there are three different types of β-receptors in humans. The heart expresses primarily β_1_-receptors and presents a relatively small proportion of β_2_-receptors. Most β_2_-receptors are present in human airway smooth muscle. All of the inhaled LABAs tested in the trials described here are highly selective β_2_-receptor agonists that mainly relax bronchial smooth muscle to improve lung function, dyspnea, hypoxia and exercise tolerance but have little direct impact on the heart. There is certain evidence that hypoxia, physical inactivity and oxidative stress increase sympathetic activity in patients with COPD [45]. Inhaled LABAs significantly reduce hypoxia and improve exercise tolerance, which down-regulate sympathetic activity, resulting in a reduction in myocardial oxygen consumption and cardiovascular events. Furthermore, certain studies have shown that the development of COPD is closely related to certain inflammatory cytokines. Compared with control subjects, the levels of TNF-α and IL-8 are elevated in the sputum of patients with COPD [46]. Moreover, patients with frequent exacerbations have higher levels of stable airway inflammatory markers, including the serum levels of IL-6 and IL-8 [47]. There is certain evidence that high levels of IL-8 and TNF-α increase the risk of cardiovascular events [48–50]. Inhaled LABAs can significantly suppress the release of IL-8 and TNF-α [51], which might be a mechanism for the reduction in cardiovascular events in patients with COPD.

In our meta-analysis, the sensitivity analysis showed that the benefit of inhaled LABAs was largely driven by the result of Calverley and colleagues [11], which contributed nearly 50% of the weight in the fixed-effects model. This may have been because this study enrolled the maximum number of COPD patients and lasted for 3 years, which allowed the researchers to assess the impact of inhaled LABAs on fatal cardiovascular events more accurately compared with other studies. However, there was still no increased risk of fatal cardiovascular events (RR 0.68, 95%CI 0.46 to 1.01, P = 0.06) after excluding this study. The impact of inhaled LABAs on fatal cardiovascular events in COPD patients still needs to be further confirmed by more long-term clinical trials.

There are certain discrepancies between the outcome of our meta-analysis and the results of previous studies. Based on observational studies, Gershon et al [52] and Au et al [53] reported that the use of inhaled LABAs was associated with a higher risk of cardiovascular events compared with not using these medications. Furthermore, an analysis conducted by Salpeter et al selected 13 single-dose and 20 longer-duration randomized, placebo-controlled trials to evaluate the cardiovascular effects of β_2_ agonists in patients with COPD and asthma [8]. The authors defined cardiovascular events as including sinus and ventricular tachycardia, syncope, atrial fibrillation, congestive heart failure, myocardial infarction, cardiac arrest and sudden death and estimated the effects of sinus tachycardia separately from those of the other events in the longer-duration trials. In the included trials, which lasted from 3 days to 1 year, β_2_ agonists increased the risk of cardiovascular events (RR 2.54, 95% CI 1.59 to 4.05), but when sinus tachycardia was excluded, there were no significant differences in major cardiovascular events (RR 1.66, 95% CI 0.76 to 3.60). There were certain limitations to this analysis. First, the clinical significance of this analysis with respect to the cardiovascular effects of inhaled LABAs on COPD is unclear because the analysis included trials that involved asthma patients and because only six longer trials of LABAs in COPD were included. Second, the authors only found a significant difference between β_2_ agonists and placebo for sinus tachycardia, which is considered to be a minor event. In contrast, we focused our attention on fatal cardiovascular events, which are more meaningful for clinical practice. Third, in the prior analysis, approximately one-half of adverse cardiac events occurred in a single trial of SABA use in asthma, which may have led to inaccurate outcomes regarding the effects of LABAs in COPD patients.

The results of our analysis are consistent with other published studies showing that inhaled LABAs have a beneficial effect on cardiovascular events in COPD patients. One retrospective case-control study found that inhaled LABAs decreased all-cause mortality in COPD (OR 0.92, 95% CI 0.88 to 0.96) and that there was a trend toward a benefit in terms of cardiovascular deaths (OR 0.97, 95% CI 0.84 to 1.11), although this effect did not reach statistical significance [54]. Furthermore, Ferguson et al[10] conducted a meta-analysis of seven trials assessing the cardiovascular safety of salmeterol and showed no increase in the risk of cardiovascular events compared with placebo (RR 1.03, 95% CI 0.8 to 1.3). Both groups had a similar incidence of cardiovascular events, including fatal events. Moreover, in addition to certain retrospective studies, several RCTs support our results [11, 55, 56], although two of these did not meet the inclusion criteria and were excluded from our meta-analysis [55, 56]. Campbell et al [55] compared formoterol with placebo for 8 weeks and found no significant differences between the two groups in terms of heart rate, ventricular premature beats, ventricular tachycardia events or supraventricular premature beats. Similarly, Nelson et al[56]estimated cardiac safety in a 12-week, multicenter, randomized, double-blind, placebo-controlled trial with formoterol. Compared with placebo, the occurrence of treatment-emergent adverse cardiac events was similar in the formoterol group, and no deaths or serious cardiac adverse events occurred during the treatment period. Furthermore, a new once-daily LABA, known as indacaterol, was recently shown to be efficient in treating COPD patients. A secondary analysis of three clinical trials provided evidence that indacaterol did not increase the risk of cardiovascular or cerebrovascular adverse events at any dose (RR 1.35, 95% CI 0.94 to 1.92) [57].

There are certain limitations to our study that make it difficult to achieve definitive conclusions. First, as with any meta-analysis, the potential for publication bias needs to be discussed. However, visual inspection of funnel plots suggested that publication bias was unlikely in this meta-analysis. Second, none of the included trials were specifically designed to monitor the risk of cardiovascular events, which might have resulted in incomplete reporting of cardiovascular events. Third, the data used in this analysis were based on the judgment of the investigators, which might result in discrepant reports between studies. Fourth, in our meta-analysis, the sensitivity analysis showed that the benefit of inhaled LABAs was largely driven by one study [11], which contributed nearly 50% of the weight in the fixed-effects model. This finding indicated that more studies with larger samples and longer durations will be needed to evaluate the association between LABAs and cardiovascular events. Finally, because of the low number of clinical trials examined, the results derived from the subgroup analyses should be interpreted with considerable caution. Despite these limitations, we believe that this analysis adds more positive evidence for the cardiovascular safety of inhaled LABA use in patients with COPD.

## Conclusions

In summary, despite certain limitations, our findings still have potential implications. We performed a meta-analysis of 24 RCTs, and the outcome indicates that inhaled LABAs do not increase the risk of fatal cardiovascular events in COPD, which was especially noted in long-term trials with severe COPD patients. Therefore, it is safe to use LABAs in COPD patients who have comorbidities consisting of cardiovascular diseases.

## Supporting Information

S1 PRISMA Checklist (DOC)

S1 Table Main characteristics of enrolled studies. (DOCX)

S2 Table The full-text excluded articles. (DOCX)
