# Supplementary material for: Inhaled Long-Acting β2-Agonists Do Not Increase Fatal Cardiovascular Adverse Events in COPD: A Meta-Analysis
Source: PLoS One. 2015 Sep 17;10(9):e0137904. doi: 10.1371/journal.pone.0137904 (PMC4574772; doi:10.1371/journal.pone.0137904)
Supplement: S1 Table — (DOCX) [file pone.0137904.s002.docx]

| Source | Intervention | Trial duration | No.of patients | Ages(SD) | Predicted FEV1(%), Mean(SD) | Jadad score |
| --- | --- | --- | --- | --- | --- | --- |
| Brusasco 2003 | SAL 50ug bid | 6 months | 1207 | 64.1(8.5) | 37.7(11.7) | 4 |
|  | TI 18ug qd |  |  | 63.8(8.0) | 39.2(11.6) |  |
|  | PL |  |  | 64.6(8.6) | 38.7(12.1) |  |
| Rennard 2001 | SAL 42ug bid | 12 weeks | 405 | 63.9(0.9) | NA | 5 |
|  | ipratropium 36ug qod |  |  | 61.7(0.8) | NA |  |
|  | PL |  |  | 63.7(0.7) | NA |  |
| Donohue 2002 | SAL 50ug bid | 6 months | 623 | 64.6(8.1) | NA | 4 |
|  | TI 18ug qd |  |  | 64.5(7.9) | NA |  |
|  | PL |  |  | 65.6(7.8) | NA |  |
| Rossi 2002 | FORM 12ug bid | 12 months | 1127 | 63 | 47 | 3 |
|  | FORM 24ug bid |  |  | 62 | 47 |  |
|  | PL |  |  | 63 | 49 |  |
|  | Theophylline 200mg bid |  |  | 64 | 46 |  |
| Baumgartner | ARF 15ug bid | 12 weeks | 717 | 62.0(9.1) | 40.2(12.4) | 4 |
| 2007 | ARF 25ug bid |  |  | 63.5(9.2) | 39.6(13.5) |  |
|  | ARF 50ug qd |  |  | 62.4(9.4) | 40.9(13.4) |  |
|  | SAL 42ug bid |  |  | 63.4(8.8) | 41.6(13.2) |  |
|  | PL |  |  | 63.1(8.4) | 40.6(12.6) |  |
| Kornmann | Indacaterol 150ug qd | 6 months | 1002 | 63(8.7) | 54.0(14.0) | 4 |
| 2011 | SAL 50ug bid |  |  | 63(9.2) | 53(13.6) |  |
|  | PL |  |  | 64(8.6) | 53(14.2) |  |
| Stockley 2006 | SAL 50ug bid | 12 months | 634 | 62.4(9.2) | 46.1(14.5) | 5 |
|  | PL |  |  | 62.3(9.1) | 45.8(14.1) |  |
| Calverley 2003 | SAL 50ug bid | 12 months | 1465 | 63.2(8.6) | 44.3(13.8) | 5 |
|  | fluticasone 50ug bid |  |  | 63.5(8.5) | 45.0(13.6) |  |
|  | SAL/fluticasone 50/500ug bid |  |  | 62.7(8.7) | 44.8(14.7) |  |
|  | PL |  |  | 63.4(8.6) | 44.2(13.7) |  |
| Chapman 2011 | Indacaterol 150ug qd | 52 weeks | 1263 | 62.5(9.52) | 53.9(13.69) | 4 |
|  | Indacaterol 300ug qd |  |  | 62.5(9.00) | 56.6(14.62) |  |
|  | PL |  |  | 62.8(9.18) | 56.3(14.83) |  |
| Ferguson 2014 | Olodaterol 5ug qd | 48 weeks | 625 | 64.0(8.6) | 48.1(15.4) | 3 |
|  | Olodaterol 10ug qd |  |  | 65.0(8.2) | 49.4(15.5) |  |
|  | PL |  |  | 65.8(8.5) | 49.1(15.4) |  |
| Ferguson 2014 | Olodaterol 5ug qd | 48 weeks | 644 | 64.7(8.1) | 49.2(14.5) | 3 |
|  | Olodaterol 10ug qd |  |  | 65.4(9.7) | 48.4(15.4) |  |
|  | PL |  |  | 63.8(8.3) | 49.1(16.7) |  |
| Koch 2014 | Olodaterol 5ug qd | 48 weeks | 906 | 63.7(9.1) | 52.3(14.9) | 3 |
|  | Olodaterol 10ug qd |  |  | 62.6(8.8) | 49.8(14.7) |  |
|  | FORM 12ug qd |  |  | 64.8(8.6) | 52.8(14.6) |  |
|  | PL |  |  | 64.0(8.4) | 50.0(14.7) |  |
| Koch 2014 | Olodaterol 5ug qd | 48 weeks | 937 | 63.7(8.8) | 52.2(14.7) | 3 |
|  | Olodaterol 10ug qd |  |  | 63.8(8.5) | 51.3(14.9) |  |
|  | FORM 12ug qd |  |  | 65.0(8.2) | 51.0(15.8) |  |
|  | PL |  |  | 63.9(7.8) | 52.0(16.0) |  |
| Dahl 2010 | Indacaterol 300ug qd | 52 weeks | 1732 | 64 | 51.5 | 5 |
|  | Indacaterol 600ug qd |  |  | 63 | 50.8 |  |
|  | FORM 12ug bid |  |  | 64 | 52.5 |  |
|  | PL |  |  | 63 | 52 |  |
| Calverley 2007 | SAL 50ug bid | 3 years | 6184 | 65.1(8.2) | 43.6(12.6) | 5 |
|  | fluticasone 500ug bid |  |  | 65.0(8.4) | 44.1(12.3) |  |
|  | SAL/fluticasone 50/500ug bid |  |  | 65.0(8.3) | 44.3(12.3) |  |
|  | PL |  |  | 65.0(8.2) | 44.1(12.3) |  |
| Kerwin 2011 | Indacaterol 75ug qd | 12 weeks | 323 | 64.0(8.3) | 54(12.8) | 5 |
|  | PL |  |  | 64.0(9.4) | 53(13.4) |  |
| Kerwin 2011 | Indacaterol 75ug qd | 12 weeks | 318 | 61.0(9.8) | 56(12.8) | 5 |
|  | PL |  |  | 62.0(9.9) | 54(13.6) |  |
| Feldman 2010 | Indacaterol 150ug qd | 12 weeks | 416 | 62.9(9.89) | 54.4(13.38) | 4 |
|  | PL |  |  | 63.2(9.62) | 55.8(14.08) |  |
| Singh 2014 | Aclidinium/formoterol 400/12ug bid | 24 weeks | 1729 | 62.7(8.1) | 54.6(13.1) | 5 |
|  | Aclidinium/formoterol 400/6ug bid |  |  | 62.9(7.7) | 54.1(13.0) |  |
|  | Aclidinium 400ug bid |  |  | 63.1(8.2) | 53.6(13.0) |  |
|  | FORM 12ug bid |  |  | 63.4(7.8) | 54.5(13.2) |  |
|  | PL |  |  | 64.2(8.0) | 55.0(13.4) |  |
| Kerwin 2013 | Fluticasone furoate/vilanterol 100/25ug qd | 24 weeks | 1030 | 62.3(8.49) | 47.8(12.28) | 4 |
|  | Fluticasone furoate/vilanterol 50/25ug qd |  |  | 62.8(9.13) | 48.4(12.66) |  |
|  | Fluticasone furoate100ug qd |  |  | 62.7(9.47) | 46.9(12.73) |  |
|  | Vilanterol 25ug qd |  |  | 63.4(9.58) | 49.9(12.05) |  |
|  | PL |  |  | 62.1(8.80) | 48.5(12.46) |  |
| Martinez 2013 | Fluticasone furoate/vilanterol 100/25ug qd | 24 weeks | 1224 | 61.9(8.79) | 48.1(12.85) | 4 |
|  | Fluticasone furoate/vilanterol 200/25ug qd |  |  | 61.1(8.58) | 47.1(12.76) |  |
|  | Fluticasone furoate 100ug qd |  |  | 61.8(8.28) | 48.4(12.17) |  |
|  | Fluticasone furoate 200ug qd |  |  | 61.8(9.02) | 47.1(11.98) |  |
|  | Vilanterol 25ug qd |  |  | 61.2(8.62) | 48.5(12.89) |  |
|  | PL |  |  | 61.9(8.14) | 48.3(12.71) |  |
| Bateman 2013 | Indacaterol/glycopyrronium 50ug qd | 26 weeks | 2144 | 64.0(8.9) | 55.7(13.2) | 5 |
|  | Indacaterol 150ug qd |  |  | 63.6(8.8) | 54.9(12.9) |  |
|  | glycopyrronium 50ug qd |  |  | 64.3(9.0) | 55.1(13.4) |  |
|  | PL |  |  | 64.4(8.6) | 55.2(12.7) |  |
| Donohue 2013 | Umeclidinium/vilanterol 62.5/25 mcg qd | 24 weeks | 1532 | 63.1(8.71) | 47.8(13.19) | 5 |
|  | Umeclidinium 62.5mcg qd |  |  | 64.0(9.16) | 46.8(13.39) |  |
|  | Vilanterol 25mcg qd |  |  | 62.7(8.52) | 48.2(13.27) |  |
|  | PL |  |  | 62.2(9.04) | 46.7(12.71) |  |
| Tashkin 2008 | Budesonide/Formoterol 160/4.5ug bid | 6 months | 1704 | 63.1(9.0) | 39.05(11.78) | 5 |
|  | Budesonide/Formoterol 80/4.5ug bid |  |  | 63.6(9.0) | 39.87(11.23) |  |
|  | FORM 4.5ug bid |  |  | 63.5(9.5) | 39.59(12.76) |  |
|  | PL |  |  | 63.2(9.6) | 41.28(12.14) |  |
|  | Budesonide 160ug bid |  |  | 63.4(8.8) | 39.72(12.01) |  |
|  | Budesonide+Formoterol 160/4.5ug bid |  |  | 63.7(9.0) | 39.15(11.41) |  |
| Donohue 2010 | Indacaterol 150ug qd | 26 weeks | 1683 | 63.4(9.40) | 56.1(14.47) | 4 |
|  | Indacaterol 300ug qd |  |  | 63.3(9.32) | 56.3(14.5) |  |
|  | TI 18ug qd |  |  | 64.0(8.77) | 53.9(15.56) |  |
|  | PL |  |  | 63.6(8.92) | 56.1(14.27) |  |
| Bogdan 2011 | FORM 4.5ug bid | 12 weeks | 613 | 66.7 | 50.4 | 4 |
|  | FORM 9ug bid |  |  | 67.2 | 51.5 |  |
|  | PL |  |  | 66.3 | 52.5 |  |
| Celli 2014 | Umeclidinium/vilanterol 125/25mcg qd | 24 weeks | 1489 | 63.4(8.08) | 47.7(12.53) | 3 |
|  | Umeclidinium 125mcg qd |  |  | 63.1(8.48) | 48.8(12.32) |  |
|  | Vilanterol 25mcg qd |  |  | 62.8(8.8) | 48.5(12.74) |  |
|  | PL |  |  | 62.2(8.53) | 47.6(12.47) |  |

S1-Table. Main characteristics of enrolled studies.
