# Supplementary material for: Inhaled Long-Acting β2-Agonists Do Not Increase Fatal Cardiovascular Adverse Events in COPD: A Meta-Analysis
Source: PLoS One. 2015 Sep 17;10(9):e0137904. doi: 10.1371/journal.pone.0137904 (PMC4574772; doi:10.1371/journal.pone.0137904)
Supplement: S2 Table — (DOCX) [file pone.0137904.s003.docx]

No fatal cardiovascular events in LABAs and placebo groups (N=12):

1. Aalbers R, Ayres J, Backer V, Decramer M, Lier PA, et al. (2002) Formoterol in patients with chronic obstructive pulmonary disease: a randomized, controlled, 3-month trial. Eur Respir J 19: 936-943.

2. Barnes PJ, Pocock SJ, Magnussen H, Iqbal A, Kramer B, et al. (2010) Integrating indacaterol dose selection in a clinical study in COPD using an adaptive seamless design. Pulm Pharmacol Ther 23: 165-171.

3. Boyd G, Morice AH, Pounsford JC, Siebert M, Peslis N, et al. (1997) An evaluation of salmeterol in the treatment of chronic obstructive pulmonary disease (COPD). Eur Respir J 10: 815-821.

4. Dahl R, Greefhorst LA, Nowak D, Nonikov V, Byrne AM, et al. (2001) Inhaled formoterol dry powder versus ipratropium bromide in chronic obstructive pulmonary disease. Am J Respir Crit Care Med 164: 778-784.

5. Gross NJ, Nelson HS, Lapidus RJ, Dunn L, Lynn L, et al. (2008) Efficacy and safety of formoterol fumarate delivered by nebulization to COPD patients. Respir Med 102: 189-197.

6. Hanania NA, Darken P, Horstman D, Reisner C, Lee B, et al. (2003) The efficacy and safety of fluticasone propionate (250 microg)/salmeterol (50 microg) combined in the Diskus inhaler for the treatment of COPD. Chest 124: 834-843.

7. Kinoshita M, Lee SH, Hang LW, Ichinose M, Hosoe M, et al. (2012) Efficacy and safety of indacaterol 150 and 300 microg in chronic obstructive pulmonary disease patients from six Asian areas including Japan: a 12-week, placebo-controlled study. Respirology 17: 379-389.

8. Mahler DA, Donohue JF, Barbee RA, Goldman MD, Gross NJ, et al. (1999) Efficacy of salmeterol xinafoate in the treatment of COPD. Chest 115: 957-965.

9. Mahler DA, Wire P, Horstman D, Chang CN, Yates J, et al. (2002) Effectiveness of fluticasone propionate and salmeterol combination delivered via the Diskus device in the treatment of chronic obstructive pulmonary disease. Am J Respir Crit Care Med 166: 1084-1091.

10. Nelson HS, Gross NJ, Levine B, Kerwin EM, Rinehart M, et al. (2007) Cardiac safety profile of nebulized formoterol in adults with COPD: a 12-week, multicenter, randomized, double- blind, double-dummy, placebo- and active-controlled trial. Clin Ther 29: 2167-2178.

11. Rennard SI, Tashkin DP, McElhattan J, Goldman M, Ramachandran S, et al. (2009) Efficacy and tolerability of budesonide/formoterol in one hydrofluoroalkane pressurized metered-dose inhaler in patients with chronic obstructive pulmonary disease: results from a 1-year randomized controlled clinical trial. Drugs 69: 549-565.

12. Yao W, Wang C, Zhong N, Han X, Wu C, et al. (2014) Effect of once-daily indacaterol in a predominantly Chinese population with chronic obstructive pulmonary disease: a 26-week Asia-Pacific study. Respirology 19: 231-238.

Not RCT about LABA vs placebo (N=36):

1. Aaron SD, Vandemheen KL, Fergusson D, Maltais F, Bourbeau J, et al. (2007) Tiotropium in combination with placebo, salmeterol, or fluticasone-salmeterol for treatment of chronic obstructive pulmonary disease: a randomized trial. Ann Intern Med 146: 545-555.

2. Agusti A, de Teresa L, De Backer W, Zvarich MT, Locantore N, et al. (2014) A comparison of the efficacy and safety of once-daily fluticasone furoate/vilanterol with twice-daily fluticasone propionate/salmeterol in moderate to very severe COPD. Eur Respir J 43: 763-772.

3. Barnes NC, Qiu YS, Pavord ID, Parker D, Davis PA, et al. (2006) Antiinflammatory effects of salmeterol/fluticasone propionate in chronic obstructive lung disease. Am J Respir Crit Care Med 173: 736-743.

4. Beeh KM, Hederer B, Glaab T, Muller A, Rutten-van Moelken M, et al. (2009) Study design considerations in a large COPD trial comparing effects of tiotropium with salmeterol on exacerbations. Int J Chron Obstruct Pulmon Dis 4: 119-125.

5. Boscia JA, Pudi KK, Zvarich MT, Sanford L, Siederer SK, et al. (2012) Effect of once-daily fluticasone furoate/vilanterol on 24-hour pulmonary function in patients with chronic obstructive pulmonary disease: a randomized, three-way, incomplete block, crossover study. Clin Ther 34: 1655-1666 e1655.

6. Bourbeau J, Christodoulopoulos P, Maltais F, Yamauchi Y, Olivenstein R, et al. (2007) Effect of salmeterol/fluticasone propionate on airway inflammation in COPD: a randomised controlled trial. Thorax 62: 938-943.

7. Buhl R, Dunn LJ, Disdier C, Lassen C, Amos C, et al. (2011) Blinded 12-week comparison of once-daily indacaterol and tiotropium in COPD. Eur Respir J 38: 797-803.

8. Chapman KR, Beeh KM, Beier J, Bateman ED, D'Urzo A, et al. (2014) A blinded evaluation of the efficacy and safety of glycopyrronium, a once-daily long-acting muscarinic antagonist, versus tiotropium, in patients with COPD: the GLOW5 study. BMC Pulm Med 14: 4.

9. Dahl R, Jadayel D, Alagappan VK, Chen H, Banerji D (2013) Efficacy and safety of QVA149 compared to the concurrent administration of its monocomponents indacaterol and glycopyrronium: the BEACON study. Int J Chron Obstruct Pulmon Dis 8: 501-508.

10. Donohue JF, Niewoehner D, Brooks J, O'Dell D, Church A (2014) Safety and tolerability of once-daily umeclidinium/vilanterol 125/25 mcg and umeclidinium 125 mcg in patients with chronic obstructive pulmonary disease: results from a 52-week, randomized, double-blind, placebo-controlled study. Respir Res 15: 78.

11. Dransfield MT, Feldman G, Korenblat P, LaForce CF, Locantore N, et al. (2014) Efficacy and safety of once-daily fluticasone furoate/vilanterol (100/25 mcg) versus twice-daily fluticasone propionate/salmeterol (250/50 mcg) in COPD patients. Respir Med 108: 1171-1179.

12. Fukuchi Y, Samoro R, Fassakhov R, Taniguchi H, Ekelund J, et al. (2013) Budesonide/formoterol via Turbuhaler(R) versus formoterol via Turbuhaler(R) in patients with moderate to severe chronic obstructive pulmonary disease: phase III multinational study results. Respirology 18: 866-873.

13. Hagedorn C, Kassner F, Banik N, Ntampakas P, Fielder K (2013) Influence of salmeterol/fluticasone via single versus separate inhalers on exacerbations in severe/very severe COPD. Respir Med 107: 542-549.

14. Hanania NA, Boota A, Kerwin E, Tomlinson L, Denis-Mize K (2009) Efficacy and safety of nebulized formoterol as add-on therapy in COPD patients receiving maintenance tiotropium bromide: Results from a 6-week, randomized, placebo-controlled, clinical trial. Drugs 69: 1205-1216.

15. Hasegawa M, Dobashi K, Horie T, Wada N, Shirakura K (2012) Influence of inhaled procaterol on pulmonary rehabilitation in chronic obstructive pulmonary disease. Respir Investig 50: 135-139.

16. Hoshino M, Ohtawa J (2014) Computed tomography assessment of airway dimensions with combined tiotropium and indacaterol therapy in COPD patients. Respirology 19: 403-410.

17. Jorup C, Bengtsson T, Strandgarden K, Sjobring U (2014) Transient paradoxical bronchospasm associated with inhalation of the LAMA AZD9164: analysis of two Phase I, randomised, double-blind, placebo-controlled studies. BMC Pulm Med 14: 52.

18. Lofdahl CG, Postma DS, Pride NB, Boe J, Thoren A (2007) Possible protection by inhaled budesonide against ischaemic cardiac events in mild COPD. Eur Respir J 29: 1115-1119.

19. Magnussen H, Disse B, Rodriguez-Roisin R, Kirsten A, Watz H, et al. (2014) Withdrawal of inhaled glucocorticoids and exacerbations of COPD. N Engl J Med 371: 1285-1294.

20. Mahler DA, D'Urzo A, Bateman ED, Ozkan SA, White T, et al. (2012) Concurrent use of indacaterol plus tiotropium in patients with COPD provides superior bronchodilation compared with tiotropium alone: a randomised, double-blind comparison. Thorax 67: 781-788.

21. Ohar JA, Crater GD, Emmett A, Ferro TJ, Morris AN, et al. (2014) Fluticasone propionate/salmeterol 250/50 mug versus salmeterol 50 mug after chronic obstructive pulmonary disease exacerbation. Respir Res 15: 105.

22. Pepin JL, Cockcroft JR, Midwinter D, Sharma S, Rubin DB, et al. (2014) Long-acting bronchodilators and arterial stiffness in patients with COPD: a comparison of fluticasone furoate/vilanterol with tiotropium. Chest 146: 1521-1530.

23. Rossi A, van der Molen T, del Olmo R, Papi A, Wehbe L, et al. (2014) INSTEAD: a randomised switch trial of indacaterol versus salmeterol/fluticasone in moderate COPD. Eur Respir J 44: 1548-1556.

24. Sharafkhaneh A, Southard JG, Goldman M, Uryniak T, Martin UJ (2012) Effect of budesonide/formoterol pMDI on COPD exacerbations: a double-blind, randomized study. Respir Med 106: 257-268.

25. Sin DD, Lacy P, York E, Man SF (2004) Effects of fluticasone on systemic markers of inflammation in chronic obstructive pulmonary disease. Am J Respir Crit Care Med 170: 760-765.

26. Singh D, Brooks J, Hagan G, Cahn A, O'Connor BJ (2008) Superiority of "triple" therapy with salmeterol/fluticasone propionate and tiotropium bromide versus individual components in moderate to severe COPD. Thorax 63: 592-598.

27. Singh D, Nicolini G, Bindi E, Corradi M, Guastalla D, et al. (2014) Extrafine beclomethasone/formoterol compared to fluticasone/salmeterol combination therapy in COPD. BMC Pulm Med 14: 43.

28. Snell N, Foster M, Vestbo J (2013) Efficacy and safety of AZD1981, a CRTH2 receptor antagonist, in patients with moderate to severe COPD. Respir Med 107: 1722-1730.

29. Verhoeven GT, Garrelds IM, Hoogsteden HC, Zijlstra FJ (2001) Effects of fluticasone propionate inhalation on levels of arachidonic acid metabolites in patients with chronic obstructive pulmonary disease. Mediators Inflamm 10: 21-26.

30. Vincken W, Aumann J, Chen H, Henley M, McBryan D, et al. (2014) Efficacy and safety of coadministration of once-daily indacaterol and glycopyrronium versus indacaterol alone in COPD patients: the GLOW6 study. Int J Chron Obstruct Pulmon Dis 9: 215-228.

31. Vogelmeier C, Fabbri LM, Rabe KF, Beeh KM, Schmidt H, et al. (2013) Effect of tiotropium vs. salmeterol on exacerbations: GOLD II and maintenance therapy naive patients. Respir Med 107: 75-83.

32. Vogelmeier C, Hederer B, Glaab T, Schmidt H, Rutten-van Molken MP, et al. (2011) Tiotropium versus salmeterol for the prevention of exacerbations of COPD. N Engl J Med 364: 1093-1103.

33. Wedzicha JA, Singh D, Vestbo J, Paggiaro PL, Jones PW, et al. (2014) Extrafine beclomethasone/formoterol in severe COPD patients with history of exacerbations. Respir Med 108: 1153-1162.

34. Wise RA, Anzueto A, Cotton D, Dahl R, Devins T, et al. (2013) Tiotropium Respimat inhaler and the risk of death in COPD. N Engl J Med 369: 1491-1501.

35. Wouters EF, Postma DS, Fokkens B, Hop WC, Prins J, et al. (2005) Withdrawal of fluticasone propionate from combined salmeterol/fluticasone treatment in patients with COPD causes immediate and sustained disease deterioration: a randomised controlled trial. Thorax 60: 480-487.

36. ZuWallack RL, Mahler DA, Reilly D, Church N, Emmett A, et al. (2001) Salmeterol plus theophylline combination therapy in the treatment of COPD. Chest 119: 1661-1670.

No report the details of cardiovascular events (N=39):

1. Anzueto A, Ferguson GT, Feldman G, Chinsky K, Seibert A, et al. (2009) Effect of fluticasone propionate/salmeterol (250/50) on COPD exacerbations and impact on patient outcomes. COPD 6: 320-329.

2. Balint B, Watz H, Amos C, Owen R, Higgins M, et al. (2010) Onset of action of indacaterol in patients with COPD: comparison with salbutamol and salmeterol-fluticasone. Int J Chron Obstruct Pulmon Dis 5: 311-318.

3. Briggs DD, Jr., Covelli H, Lapidus R, Bhattycharya S, Kesten S, et al. (2005) Improved daytime spirometric efficacy of tiotropium compared with salmeterol in patients with COPD. Pulm Pharmacol Ther 18: 397-404.

4. Calverley PM, Boonsawat W, Cseke Z, Zhong N, Peterson S, et al. (2003) Maintenance therapy with budesonide and formoterol in chronic obstructive pulmonary disease. Eur Respir J 22: 912-919.

5. Calverley PM, Kuna P, Monso E, Costantini M, Petruzzelli S, et al. (2010) Beclomethasone/formoterol in the management of COPD: a randomised controlled trial. Respir Med 104: 1858-1868.

6. Campbell M, Eliraz A, Johansson G, Tornling G, Nihlen U, et al. (2005) Formoterol for maintenance and as-needed treatment of chronic obstructive pulmonary disease. Respir Med 99: 1511-1520.

7. Cazzola M, Di Lorenzo G, Di Perna F, Calderaro F, Testi R, et al. (2000) Additive effects of salmeterol and fluticasone or theophylline in COPD. Chest 118: 1576-1581.

8. Cazzola M, Matera MG, D'Amato M, Califano C, Sanduzzi A, et al. (2003) Bronchodilator response to formoterol Turbuhaler in patients with COPD under regular treatment with formoterol Turbuhaler. Pulm Pharmacol Ther 16: 105-109.

9. Celli B, Halpin D, Hepburn R, Byrne N, Keating ET, et al. (2003) Symptoms are an important outcome in chronic obstructive pulmonary disease clinical trials: results of a 3-month comparative study using the Breathlessness, Cough and Sputum Scale (BCSS). Respir Med 97 Suppl A: S35-43.

10. Chapman KR, Arvidsson P, Chuchalin AG, Dhillon DP, Faurschou P, et al. (2002) The addition of salmeterol 50 microg bid to anticholinergic treatment in patients with COPD: a randomized, placebo controlled trial. Chronic obstructive pulmonary disease. Can Respir J 9: 178-185.

11. Dal Negro RW, Pomari C, Tognella S, Micheletto C (2003) Salmeterol & fluticasone 50 microg/250 microg bid in combination provides a better long-term control than salmeterol 50 microg bid alone and placebo in COPD patients already treated with theophylline. Pulm Pharmacol Ther 16: 241-246.

12. Donohue JF, Menjoge S, Kesten S (2003) Tolerance to bronchodilating effects of salmeterol in COPD. Respir Med 97: 1014-1020.

13. Fuso L, Incalzi RA, Basso S, Spadaro S, Tramaglino LM, et al. (2003) Effects of formoterol inhaled dry powder on exercise performance in chronic obstructive pulmonary disease: a single-center, randomized, double-blind, placebo-controlled, crossover study. Curr Ther Res Clin Exp 64: 317-326.

14. Hanrahan JP, Hanania NA, Calhoun WJ, Sahn SA, Sciarappa K, et al. (2008) Effect of nebulized arformoterol on airway function in COPD: results from two randomized trials. COPD 5: 25-34.

15. Hogan TJ, Geddes R, Gonzalez ER (2003) An economic assessment of inhaled formoterol dry powder versus ipratropium bromide pressurized metered dose inhaler in the treatment of chronic obstructive pulmonary disease. Clin Ther 25: 285-297.

16. Jones PW, Wilson K, Sondhi S (2003) Cost-effectiveness of salmeterol in patients with chronic obstructive pulmonary disease: an economic evaluation. Respir Med 97: 20-26.

17. Kardos P, Wencker M, Glaab T, Vogelmeier C (2007) Impact of salmeterol/fluticasone propionate versus salmeterol on exacerbations in severe chronic obstructive pulmonary disease. Am J Respir Crit Care Med 175: 144-149.

18. Kuna P, Ivanov Y, Trofimov VI, Saito T, Beckman O, et al. (2013) Efficacy and safety of AZD3199 vs formoterol in COPD: a randomized, double-blind study. Respir Res 14: 64.

19. Lofdahl CG, Ericsson A, Svensson K, Andreasson E (2005) Cost effectiveness of budesonide/formoterol in a single inhaler for COPD compared with each monocomponent used alone. Pharmacoeconomics 23: 365-375.

20. Man WD, Mustfa N, Nikoletou D, Kaul S, Hart N, et al. (2004) Effect of salmeterol on respiratory muscle activity during exercise in poorly reversible COPD. Thorax 59: 471-476.

21. Mansori F, Nemat Khorasani A, Boskabady MH, Boskabady M (2010) The effect of inhaled salmeterol, alone and in combination with fluticasone propionate, on management of COPD patients. Clin Respir J 4: 241-247.

22. Mroz RM, Minarowski L, Chyczewska E (2013) Indacaterol add-on therapy improves lung function, exercise capacity and life quality of COPD patients. Adv Exp Med Biol 756: 23-28.

23. Najafzadeh M, Marra CA, Sadatsafavi M, Aaron SD, Sullivan SD, et al. (2008) Cost effectiveness of therapy with combinations of long acting bronchodilators and inhaled steroids for treatment of COPD. Thorax 63: 962-967.

24. O'Donnell DE, Sciurba F, Celli B, Mahler DA, Webb KA, et al. (2006) Effect of fluticasone propionate/salmeterol on lung hyperinflation and exercise endurance in COPD. Chest 130: 647-656.

25. Sin DD, Man SF, Marciniuk DD, Ford G, FitzGerald M, et al. (2006) Can inhaled fluticasone alone or in combination with salmeterol reduce systemic inflammation in chronic obstructive pulmonary disease? Study protocol for a randomized controlled trial [NCT00120978]. BMC Pulm Med 6: 3.

26. Singh D, Kampschulte J, Wedzicha JA, Jones PW, Cohuet G, et al. (2013) A trial of beclomethasone/formoterol in COPD using EXACT-PRO to measure exacerbations. Eur Respir J 41: 12-17.

27. Soriano JB, Vestbo J, Pride NB, Kiri V, Maden C, et al. (2002) Survival in COPD patients after regular use of fluticasone propionate and salmeterol in general practice. Eur Respir J 20: 819-825.

28. Szafranski W, Cukier A, Ramirez A, Menga G, Sansores R, et al. (2003) Efficacy and safety of budesonide/formoterol in the management of chronic obstructive pulmonary disease. Eur Respir J 21: 74-81.

29. Tashkin DP, Pearle J, Iezzoni D, Varghese ST (2009) Formoterol and tiotropium compared with tiotropium alone for treatment of COPD. COPD 6: 17-25.

30. Terzano C, Petroianni A, Conti V, Ceccarelli D, Graziani E, et al. (2008) Rational timing of combination therapy with tiotropium and formoterol in moderate and severe COPD. Respir Med 102: 1701-1707.

31. Ulrik CS (1995) Efficacy of inhaled salmeterol in the management of smokers with chronic obstructive pulmonary disease: a single centre randomised, double blind, placebo controlled, crossover study. Thorax 50: 750-754.

32. van Noord JA, Buhl R, Laforce C, Martin C, Jones F, et al. (2010) QVA149 demonstrates superior bronchodilation compared with indacaterol or placebo in patients with chronic obstructive pulmonary disease. Thorax 65: 1086-1091.

33. van Noord JA, de Munck DR, Bantje TA, Hop WC, Akveld ML, et al. (2000) Long-term treatment of chronic obstructive pulmonary disease with salmeterol and the additive effect of ipratropium. Eur Respir J 15: 878-885.

34. Vestbo J, Pauwels R, Anderson JA, Jones P, Calverley P, et al. (2005) Early onset of effect of salmeterol and fluticasone propionate in chronic obstructive pulmonary disease. Thorax 60: 301-304.

35. Vogelmeier C, Kardos P, Harari S, Gans SJ, Stenglein S, et al. (2008) Formoterol mono- and combination therapy with tiotropium in patients with COPD: a 6-month study. Respir Med 102: 1511-1520.

36. Vogelmeier C, Ramos-Barbon D, Jack D, Piggott S, Owen R, et al. (2010) Indacaterol provides 24-hour bronchodilation in COPD: a placebo-controlled blinded comparison with tiotropium. Respir Res 11: 135.

37. Wadbo M, Lofdahl CG, Larsson K, Skoogh BE, Tornling G, et al. (2002) Effects of formoterol and ipratropium bromide in COPD: a 3-month placebo-controlled study. Eur Respir J 20: 1138-1146.

38. Wedzicha JA, Calverley PM, Seemungal TA, Hagan G, Ansari Z, et al. (2008) The prevention of chronic obstructive pulmonary disease exacerbations by salmeterol/fluticasone propionate or tiotropium bromide. Am J Respir Crit Care Med 177: 19-26.

39. Worth H, Forster K, Eriksson G, Nihlen U, Peterson S, et al. (2010) Budesonide added to formoterol contributes to improved exercise tolerance in patients with COPD. Respir Med 104: 1450-1459.

Not randomized controlled trials last for 3 months or parallel-group study (N=32):

1. Bateman ED, van Dyk M, Sagriotis A (2008) Comparable spirometric efficacy of tiotropium compared with salmeterol plus fluticasone in patients with COPD: a pilot study. Pulm Pharmacol Ther 21: 20-25.

2. Beeh KM, Korn S, Beier J, Jadayel D, Henley M, et al. (2014) Effect of QVA149 on lung volumes and exercise tolerance in COPD patients: the BRIGHT study. Respir Med 108: 584-592.

3. Beier J, Chanez P, Martinot JB, Schreurs AJ, Tkacova R, et al. (2007) Safety, tolerability and efficacy of indacaterol, a novel once-daily beta(2)-agonist, in patients with COPD: a 28-day randomised, placebo controlled clinical trial. Pulm Pharmacol Ther 20: 740-749.

4. Beier J, Kirsten AM, Mroz R, Segarra R, Chuecos F, et al. (2013) Efficacy and safety of aclidinium bromide compared with placebo and tiotropium in patients with moderate-to-severe chronic obstructive pulmonary disease: results from a 6-week, randomized, controlled Phase IIIb study. COPD 10: 511-522.

5. Campbell SC, Criner GJ, Levine BE, Simon SJ, Smith JS, et al. (2007) Cardiac safety of formoterol 12 microg twice daily in patients with chronic obstructive pulmonary disease. Pulm Pharmacol Ther 20: 571-579.

6. Canto ND, Ribeiro JP, Neder JA, Chiappa GR (2012) Addition of tiotropium to formoterol improves inspiratory muscle strength after exercise in COPD. Respir Med 106: 1404-1412.

7. Cazzola M, Di Perna F, Centanni S, Califano C, Donner CF, et al. (1999) Acute effect of pretreatment with single conventional dose of salmeterol on dose-response curve to oxitropium bromide in chronic obstructive pulmonary disease. Thorax 54: 1083-1086.

8. Cazzola M, Noschese P, Salzillo A, De Giglio C, D'Amato G, et al. (2005) Bronchodilator response to formoterol after regular tiotropium or to tiotropium after regular formoterol in COPD patients. Respir Med 99: 524-528.

9. Cazzola M, Santus P, Di Marco F, Carlucci P, Mondoni M, et al. (2004) Onset of action of formoterol/budesonide in single inhaler vs. formoterol in patients with COPD. Pulm Pharmacol Ther 17: 121-125.

10. Feldman G, Walker RR, Brooks J, Mehta R, Crater G (2012) 28-Day safety and tolerability of umeclidinium in combination with vilanterol in COPD: a randomized placebo-controlled trial. Pulm Pharmacol Ther 25: 465-471.

11. Feldman GJ, Bernstein JA, Hamilton A, Nivens MC, Korducki L, et al. (2014) The 24-h FEV1 time profile of olodaterol once daily via Respimat(R) and formoterol twice daily via Aerolizer(R) in patients with GOLD 2-4 COPD: results from two 6-week crossover studies. Springerplus 3: 419.

12. Grove A, Lipworth BJ, Reid P, Smith RP, Ramage L, et al. (1996) Effects of regular salmeterol on lung function and exercise capacity in patients with chronic obstructive airways disease. Thorax 51: 689-693.

13. Hanania NA, Feldman G, Zachgo W, Shim JJ, Crim C, et al. (2012) The efficacy and safety of the novel long-acting beta2 agonist vilanterol in patients with COPD: a randomized placebo-controlled trial. Chest 142: 119-127.

14. Kato M, Makita H, Uemura K, Fukuchi Y, Hosoe M, et al. (2010) Bronchodilator efficacy of single doses of indacaterol in Japanese patients with COPD: A randomised, double-blind, placebo-controlled trial. Allergol Int 59: 285-293.

15. Laforce C, Aumann J, de Teresa Parreno L, Iqbal A, Young D, et al. (2011) Sustained 24-hour efficacy of once daily indacaterol (300 mug) in patients with chronic obstructive pulmonary disease: a randomized, crossover study. Pulm Pharmacol Ther 24: 162-168.

16. Maltais F, Singh S, Donald AC, Crater G, Church A, et al. (2014) Effects of a combination of umeclidinium/vilanterol on exercise endurance in patients with chronic obstructive pulmonary disease: two randomized, double-blind clinical trials. Ther Adv Respir Dis 8: 169-181.

17. Minakata Y, Iijima H, Takahashi T, Miura M, Ogawa H, et al. (2008) Efficacy and safety of formoterol in Japanese patients with COPD. Intern Med 47: 217-223.

18. Molimard M, Bourcereau J, Le Gros V, Bourdeix I (2005) Total reversibility testing as indicator of the clinical efficacy of formoterol in COPD. Respir Med 99: 695-702.

19. Neder JA, Fuld JP, Overend T, Thirlwell J, Carter R, et al. (2007) Effects of formoterol on exercise tolerance in severely disabled patients with COPD. Respir Med 101: 2056-2064.

20. O'Donnell DE, Casaburi R, Vincken W, Puente-Maestu L, Swales J, et al. (2011) Effect of indacaterol on exercise endurance and lung hyperinflation in COPD. Respir Med 105: 1030-1036.

21. O'Donnell DE, Voduc N, Fitzpatrick M, Webb KA (2004) Effect of salmeterol on the ventilatory response to exercise in chronic obstructive pulmonary disease. Eur Respir J 24: 86-94.

22. Quinn D, Seale JP, Reisner C, Fischer T, Golden M, et al. (2014) A randomized study of formoterol fumarate in a porous particle metered-dose inhaler in patients with moderate-to-severe COPD. Respir Med 108: 1327-1335.

23. Rennard S, Bantje T, Centanni S, Chanez P, Chuchalin A, et al. (2008) A dose-ranging study of indacaterol in obstructive airways disease, with a tiotropium comparison. Respir Med 102: 1033-1044.

24. Rosenkranz B, Rouzier R, Kruse M, Dobson C, Ayre G, et al. (2006) Safety and tolerability of high-dose formoterol (via Aerolizer) and salbutamol in patients with chronic obstructive pulmonary disease. Respir Med 100: 666-672.

25. Salvi S, Brashier B, Gothi D, Karkhanis V, Madas S, et al. (2014) Bronchodilator efficacy of tiotropium-formoterol via single pressurized meter dose inhaler (pMDI) versus tiotropium alone in COPD. Pulm Pharmacol Ther 27: 90-95.

26. Singh D, Magnussen H, Kirsten A, Mindt S, Caracta C, et al. (2012) A randomised, placebo- and active-controlled dose-finding study of aclidinium bromide administered twice a day in COPD patients. Pulm Pharmacol Ther 25: 248-253.

27. Tashkin DP, Littner M, Andrews CP, Tomlinson L, Rinehart M, et al. (2008) Concomitant treatment with nebulized formoterol and tiotropium in subjects with COPD: a placebo-controlled trial. Respir Med 102: 479-487.

28. Tsagaraki V, Amfilochiou A, Markantonis SL (2006) Evidence of tachyphylaxis associated with salmeterol treatment of chronic obstructive pulmonary disease patients. Int J Clin Pract 60: 415-421.

29. van Noord JA, Aumann JL, Janssens E, Smeets JJ, Verhaert J, et al. (2005) Comparison of tiotropium once daily, formoterol twice daily and both combined once daily in patients with COPD. Eur Respir J 26: 214-222.

30. van Noord JA, Aumann JL, Janssens E, Smeets JJ, Zaagsma J, et al. (2010) Combining tiotropium and salmeterol in COPD: Effects on airflow obstruction and symptoms. Respir Med 104: 995-1004.

31. Watz H, Krippner F, Kirsten A, Magnussen H, Vogelmeier C (2014) Indacaterol improves lung hyperinflation and physical activity in patients with moderate chronic obstructive pulmonary disease--a randomized, multicenter, double-blind, placebo-controlled study. BMC Pulm Med 14: 158.

32. Wielders PL, Ludwig-Sengpiel A, Locantore N, Baggen S, Chan R, et al. (2013) A new class of bronchodilator improves lung function in COPD: a trial with GSK961081. Eur Respir J 42: 972-981.

Not RCT (N=15):

1. Calverley PM, Anderson JA, Celli B, Ferguson GT, Jenkins C, et al. (2010) Cardiovascular events in patients with COPD: TORCH study results. Thorax 65: 719-725.

2. Celli BR, Tashkin DP, Rennard SI, McElhattan J, Martin UJ (2011) Bronchodilator responsiveness and onset of effect with budesonide/formoterol pMDI in COPD. Respir Med 105: 1176-1188.

3. Decramer M, Rossi A, Lawrence D, McBryan D (2012) Indacaterol therapy in patients with COPD not receiving other maintenance treatment. Respir Med 106: 1706-1714.

4. Drivenes E, Ostrem A, Melbye H (2014) Predictors of ICS/LABA prescribing in COPD patients: a study from general practice. BMC Fam Pract 15: 42.

5. Hanania NA, Kalberg C, Yates J, Emmett A, Horstman D, et al. (2005) The bronchodilator response to salmeterol is maintained with regular, long-term use in patients with COPD. Pulm Pharmacol Ther 18: 19-22.

6. Hodder R, Kesten S, Menjoge S, Viel K (2007) Outcomes in COPD patients receiving tiotropium or salmeterol plus treatment with inhaled corticosteroids. Int J Chron Obstruct Pulmon Dis 2: 157-167.

7. Hoogendoorn M, Al MJ, Beeh KM, Bowles D, Graf von der Schulenburg JM, et al. (2013) Cost-effectiveness of tiotropium versus salmeterol: the POET-COPD trial. Eur Respir J 41: 556-564.

8. Jones PW, Anderson JA, Calverley PM, Celli BR, Ferguson GT, et al. (2011) Health status in the TORCH study of COPD: treatment efficacy and other determinants of change. Respir Res 12: 71.

9. Jones PW, Mahler DA, Gale R, Owen R, Kramer B (2011) Profiling the effects of indacaterol on dyspnoea and health status in patients with COPD. Respir Med 105: 892-899.

10. Mahler DA, Buhl R, Lawrence D, McBryan D (2013) Efficacy and safety of indacaterol and tiotropium in COPD patients according to dyspnoea severity. Pulm Pharmacol Ther 26: 348-355.

11. McGarvey LP, Magder S, Burkhart D, Kesten S, Liu D, et al. (2012) Cause-specific mortality adjudication in the UPLIFT(R) COPD trial: findings and recommendations. Respir Med 106: 515-521.

12. Ohno T, Wada S, Hanada S, Sawaguchi H, Muraki M, et al. (2014) Efficacy of indacaterol on quality of life and pulmonary function in patients with COPD and inhaler device preferences. Int J Chron Obstruct Pulmon Dis 9: 107-114.

13. Price D, Keininger D, Costa-Scharplatz M, Mezzi K, Dimova M, et al. (2014) Cost-effectiveness of the LABA/LAMA dual bronchodilator indacaterol/glycopyrronium in a Swedish healthcare setting. Respir Med 108: 1786-1793.

14. Vestbo J, Anderson J, Brook RD, Calverley PM, Celli BR, et al. (2013) The Study to Understand Mortality and Morbidity in COPD (SUMMIT) study protocol. Eur Respir J 41: 1017-1022.

15. Worth H, Chung KF, Felser JM, Hu H, Rueegg P (2011) Cardio- and cerebrovascular safety of indacaterol vs formoterol, salmeterol, tiotropium and placebo in COPD. Respir Med 105: 571-579.

Redundant data (N=2):

1. Gotfried MH, Kerwin EM, Lawrence D, Lassen C, Kramer B (2012) Efficacy of indacaterol 75 mug once-daily on dyspnea and health status: results of two double-blind, placebo-controlled 12-week studies. COPD 9: 629-636.

2. To Y, Kinoshita M, Lee SH, Hang LW, Ichinose M, et al. (2012) Assessing efficacy of indacaterol in moderate and severe COPD patients: a 12-week study in an Asian population. Respir Med 106: 1715-1721.
